# Global Coupling and Phase Locking in Laser Diode Arrays: A Review of Talbot Cavity Research

**DOI:** 10.3390/mi17080896

**Published:** 2026-07-26

**Authors:** Yikun Yang, Chenyao Huang, Jie Chen, Yixian Xie, Yuying Feng, Xi Cao, Zhengjie Guo, Fuyueyang Tan, Chuanjie Xin, Zaijin Li, Yi Qu, Lin Li

**Affiliations:** 1 College of Physics and Electronic Engineering, Hainan Normal University, Haikou 571158, China; 202513085408014@hainnu.edu.cn (Y.Y.); 202513085408004@hainnu.edu.cn (C.H.); 202413085408002@hainnu.edu.cn (J.C.); 202513085408013@hainnu.edu.cn (Y.X.); 202513085408002@hainnu.edu.cn (Y.F.); 202513085401001@hainnu.edu.cn (X.C.); 202413085408004@hainnu.edu.cn (Z.G.); 202413085408012@hainnu.edu.cn (F.T.); 202423160816@hainnu.edu.cn (C.X.); quyi@hainnu.edu.cn (Y.Q.); lin.li@hainnu.edu.cn (L.L.); 2 Hainan Provincial Key Laboratory of Laser Technology and Optoelectronic Functional Materials, Haikou 571158, China; 3 Hainan International Joint Research Center for Semiconductor Lasers, Hainan Normal University, Haikou 571158, China; 4 Collaborative Innovation Center for Flexible Talent Introduction, Haikou 571158, China

**Keywords:** phase-locked laser diode array, talbot cavity, phase locking

## Abstract

High-power semiconductor laser diode arrays (LDAs) are pivotal for applications such as optical pumping, industrial manufacturing, and precision measurement, yet they face inherent bottlenecks in balancing high output power, superior beam quality, and stable phase synchronization. The Talbot cavity, leveraging the Talbot self-imaging effect, has emerged as a core external cavity technology to address these challenges, enabling global coupling and passive phase locking of LDAs. This paper systematically reviews the research progress of Talbot cavities in phase-locked LDAs under global coupling. It elaborates on the fundamental principle of Talbot-effect-based phase locking, along with the structural characteristics and working mechanisms of three typical Talbot cavity configurations: conventional Talbot cavities, V-shaped Littrow–Talbot cavities, and monolithic integrated Talbot cavities. Furthermore, it summarizes key experimental advancements of LDAs, covering diverse laser media (e.g., near-infrared, blue, terahertz, and mid-infrared antimonide lasers) and array scales ranging from a few to thousands of emitters, with representative performance metrics including far-field visibility up to 99%, narrowed spectral linewidths achieving 20–50 pm for blue LDA, and output power exceeding 200 W. Numerical simulation progress on supermodel stability and parameter optimization is also discussed. Finally, the current challenges, such as thermal crosstalk and integration complexity, are analyzed, and future prospects involving intelligent control and novel physical mechanisms are outlined. This review aims to provide a comprehensive reference for the further development and practical application of high-brightness phase-locked laser sources.

## 1. Introduction

In 1962, R. N. Hall et al. [1] successfully developed a gallium arsenide laser diode (LD), which, for the first time, experimentally confirmed the possibility of semiconductor electrical injection lasing, marking the official birth of the semiconductor laser field. After more than 60 years of development, the manufacturing process of semiconductor lasers has gradually matured and improved from the early simple homojunction structure. The types of devices have also expanded from a single pulse working mode to a large system covering continuous, high-power, single-mode, narrow linewidth and other types. It plays an important role in the fields of pump source [2], optical communication [3], optical sensing [4], precision measurement [5], industrial manufacturing [6] and so on. However, the output power of the traditional single-tube laser is difficult to be greatly improved due to the limitation of the cavity surface damage threshold. Since the 1980s, people began to study LDAs, hoping to achieve greater output power through the advantages of the number of lasers. As the core development direction of high-power semiconductor laser technology, the LDA integrates multiple laser emission units on a single epitaxial substrate to achieve the synergistic improvement of output power and beam performance [7,8]. It also inherits the inherent advantages of traditional semiconductor lasers, such as compact size, high electro-optical conversion efficiency, excellent reliability and wide wavelength coverage [9,10,11,12]. Since the advent of semiconductor lasers, limited by the thermal accumulation, optical catastrophic damage, and output power bottlenecks of single-tube devices, researchers have gradually shifted to the idea of array integration, which has promoted the continuous evolution of its technology for decades. Early studies mostly focused on the configuration design and epitaxial process optimization of one-dimensional edge-emitting laser arrays, focusing on solving basic problems such as mode competition between units, thermal crosstalk and beam quality degradation. With the improvement of material growth technology and micro-nano processing level, the vertical cavity surface emitting laser (VCSEL) array has gradually realized large-scale preparation, and the research focus has shifted from simple power improvement to two-dimensional integration, high-speed modulation and beam shaping. In recent years, key technologies such as coherent beam combining, wavelength beam combining and intelligent thermal management have been continuously broken through [13,14,15], further promoting the development of LDAs from discrete devices to high-brightness and high-integration systems and gradually breaking through the limitations of traditional application scenarios. Despite significant progress, this field still faces many technical bottlenecks and challenges. The strong thermal coupling and thermal crosstalk between array elements under high-power operation will significantly reduce the life and working stability of the device and restrict its continuous high-power long-term reliable output. The mode competition, phase mismatch and far-field beam quality degradation caused by multi-unit parallel operation make it difficult to balance high brightness and high power. In addition, the uniformity of epitaxial materials, precise control of microcavity structure and process consistency in large-scale integration also directly affect the performance repeatability and large-scale application cost of array devices. How to improve the output power while optimizing the beam quality, achieve efficient thermal management and improve the cooperative working characteristics of the array units has become a core problem that restricts the expansion of LDAs to higher brightness, higher reliability and wider application scenarios.

This paper mainly reviews the commonly used external cavity types and research progress in phase-locked LDAs under global coupling. The structure and working principle of Talbot cavity, V-shaped Littrow–Talbot cavity and monolithic integrated Talbot cavity are introduced. Then, the performance indicators are summarized, focusing on key parameters such as far-field visibility, beam quality, output power, and number of lasers participating in phase locking.

## 2. The Basic Principle of Phase-Locked Laser Diode Array

**Figure 1:**
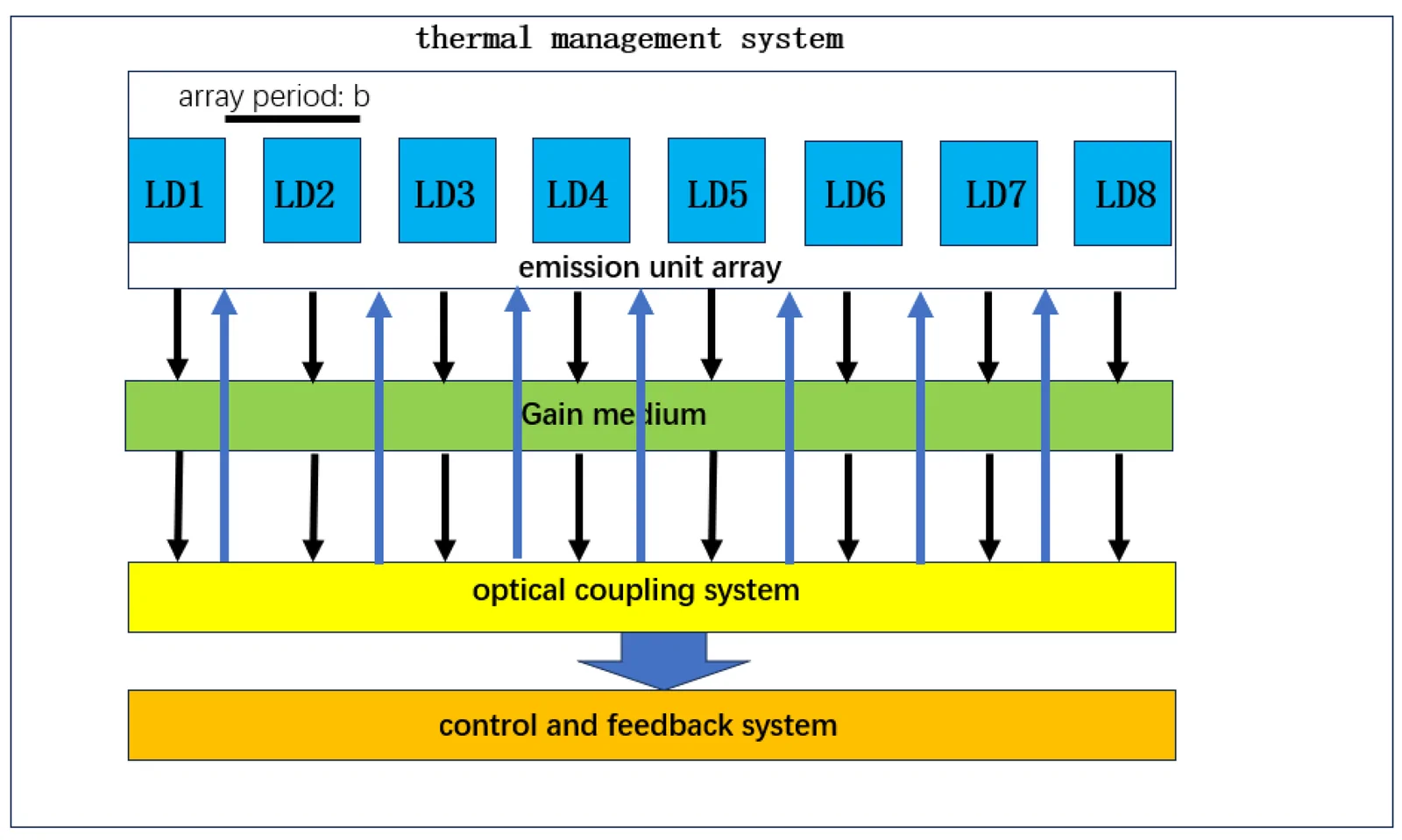
Basic structure of one-dimensional linear laser array.

The basic structure of the laser array is mainly composed of five parts: emission unit array, gain medium, thermal management system, optical coupling system and control and feedback system [16]. The emission unit array usually uses semiconductor laser diodes, and its arrangement includes one-dimensional linear array, two-dimensional stacked array and two-dimensional rectangular arrangement. The gain medium provides the condition for population inversion, transforming the medium from a light absorber into an optical amplifier, thereby amplifying light. According to the application requirements, the gain medium can be selected as inorganic liquid, solid crystal or colloidal quantum dot material. In order to control the performance degradation caused by thermal effects, the array integrates thermal management components, such as microchannel heat sinks, conductive cooling structures, or liquid circulation systems. The light emitted from the laser reaches the optical coupling system through the gain medium, and some light will be introduced into the adjacent laser, thereby changing its phase until all the lasers achieve phase synchronization. Then, the optical coupling system realizes efficient coupling through system parameter adjustment and beam propagation theory analysis. The control and feedback system achieves compact internal phase locking and large-scale expansion of all-fiber coherent laser arrays by assigning phase measurements to a series of internal Mach–Zehnder interferometers and using the gradient descent algorithm to lock the phase of each loop [17,18,19,20]. The above structure provides the basis for the high-power, high-efficiency and high-stability operation of the laser array through the photoelectric–thermal–mechanical multi-physical field collaborative design. The basic structure of the one-dimensional linear laser array is shown in Figure 1.

When the LDA is not locked, the phase of each unit is random, the far-field divergence angle is large, and the beam quality is poor, which is difficult to meet the requirements of high brightness applications. The Talbot self-imaging effect is the core of the external cavity diffraction phase-locked system, and the phase synchronization of the unit is realized by combining the external cavity optical feedback. It is the key technology to solve the coherent output of the LDA. The core mechanism can be summarized as follows: in the external cavity diffraction phase-locked system, the LDA is linearly arranged with equal spacing, and a single-laser diode unit works in the base transverse mode. The near-field light field is approximately Gaussian distribution [21], and the expression is:

(1) $$E(x,y,0)=E_{0}exp\left(-\frac{x^{2}}{\omega_{0x}^{2}}-\frac{y^{2}}{\omega_{0y}^{2}}\right)$$

where $E_{0}$ is the amplitude of the light field and $\omega_{0x}$ and $\omega_{0y}$ are the beam waist radii of Gaussian beams in x and y directions, respectively; assuming that the array contains N elements, the spacing is d, and the near-field light field is the superposition of each element when the phase is not locked, that is:

(2) $$E(x,y,0)=E_{0}\sum_{n=0}^{N-1}exp\left(-\frac{(x-nd{)}^{2}}{\omega_{0x}^{2}}-\frac{y^{2}}{\omega_{0y}^{2}}\right)exp\left(j\phi_{n}\right)$$

In the formula, $\phi_{n}$ is the random phase of the element, which leads to the non-coherent superposition of the far field and the poor beam quality. The phase locking is realized by the external cavity diffraction coupling.

The Talbot self-imaging effect is the core of the external cavity diffraction phase locking. The periodic light field is diffracted by free space, and the light field is reproduced at a specific distance. According to the scalar diffraction theory, after the plane wave is diffracted by the periodic grating, the light field at the transmission distance z is solved by the Fresnel diffraction integral. The integral form is as follows [22]:

(3) $$E(x,z)=\frac{exp(jkz)}{jkz}\int_{-\infty }^{+\infty }E(x^{\prime },0)exp\left(j\frac{k}{2z}{\left(x-x^{\prime }\right)}^{2}\right){dx}^{\prime }$$

where k = 2π/λ is the wavenumber and λ is the laser wavelength; for the light field with a period of d, the Talbot distance is $Z_{T}\;=\;2$ md^2^/λ (m is a positive integer). When transmitted to $Z_{T}$, the interference superposition effect of the diffracted light of each unit of the array just offsets the diffraction distortion, and the lateral period and spot distribution of the light field are completely consistent with the exit end face of the laser. At this time, the return light field fed back by the external cavity mirror can be accurately coupled to the active region of the corresponding laser unit. The overall light field overlap of the array is the highest, and the coupling loss is the smallest, which provides the optimal resonance condition for the global coherent coupling of the array.

In addition to the integer Talbot distance, the actual external cavity length will also be selected as the fractional Talbot distance. When the external cavity length is set to 1/2 Talbot distance, the diffraction self-imaging of the periodic light field will have a lateral half-period shift of 1/2 d, and the diffraction light field will produce a fixed additional phase mutation. At this time, in some states (in-phase), the feedback light field is misplaced with the original laser unit and cannot be effectively coupled back to the active region. The coupling loss is extremely large, the resonance threshold is high, and it is difficult to start. In some states (anti-phase), the feedback light field can achieve accurate self-imaging, the feedback coupling efficiency is the highest, the loss is the lowest, and the system is spontaneously locked in the reverse phase oscillation state. Therefore, the 1/2 Talbot distance is often used to suppress high-order stray modes and achieve stable oscillation of a single supermodel of the array. When the length of the external cavity is set to 1/4 Talbot distance, the light field exhibits a uniform coupling characteristic of multi-beam overlapping, and there is no obvious mode loss difference, which can weaken the phase-locked distortion caused by the uneven amplitude of the unit and the machining error of the device. Therefore, it is mostly used in the large-scale in-phase lock-in scene of two-dimensional array, which is suitable for the coherent combination system of a large-scale laser array.

The ideal Talbot distance can bring about the strongest diffraction coupling between array elements. However, the system can tolerate limited positional deviations. Early experimental studies have shown that the supermode selection mechanism of the Talbot cavity is highly sensitive to transmission distance; deviation from the nominal distance reduces the mode discrimination capability and the feedback strength between elements, limiting the allowable range of distance misalignment [23]. Therefore, the mechanical positioning accuracy needs to meet corresponding requirements to maintain coherent operation. However, there is currently a lack of specific experimental data for quantitative analysis of positional tolerance under different array sizes and supermode working conditions, which is a direction worthy of further research in the future.

The external cavity system consists of an LDA, an external cavity mirror and a free space transmission section. The core is to establish a coherent connection between the units through feedback and diffraction coupling. Set the external cavity length L = $Z_{T}$; the array light field is transmitted to the mirror and reproduced, reflected back to the active area of the array and superimposed with the output light field of the unit to form a coupled light field. The coupling coefficient is as follows:

(4) $$\kappa \equiv \frac{1}{E_{0}^{2}}\int_{-\infty }^{+\infty }E(x)E(x,0)dx$$

In order to describe the coupling strength, the filling factor of the array has an important influence on it. The filling factor is defined as the ratio of the emission unit to the array period. When the filling factor is moderate, the unit diffraction light field overlaps sufficiently, the coupling coefficient is high, and the phase-locked state is stable. If the filling factor is too large, the unit light field overlaps excessively, which is easy to excite high-order stray modes, resulting in multi-mode coexistence and phase-locked instability. If the filling factor is too small, the diffraction coupling strength is insufficient, the frequency traction ability is weakened, and it is difficult to achieve global coherent phase locking. The Talbot effect makes the feedback light field and the near-field light field highly coincident, and the κ is large. The external cavity provides a common resonant cavity for the array, breaking the independent lasing state of the unit and laying the foundation for phase synchronization [24].

The phase-locked core is to pull the resonant frequency of each unit to the same value and form a fixed phase difference through the external cavity coupling. Assuming that the natural frequency of the nth unit is ${\;\omega }_{n}$, the frequency ω after traction satisfies the frequency traction equation:

(5) $$\omega -\omega_{n}=\frac{\kappa }{2\tau }\sin\left(\Delta \phi_{n}\right)$$

where τ is the photon lifetime and $\Delta \phi_{n}=\phi -\phi_{n}$ is the phase difference. In the stable phase-locked state, all laser units are pulled to the same common oscillation frequency ω, and the frequency difference between each unit and the common frequency tends to zero. At this time, the phase difference between the units remains constant, which is mainly divided into the in-phase locking of the phase difference $\Delta \phi_{n}$ = 0 between adjacent units and the anti-phase locking of $\Delta \phi_{n}$ = π, in which the in-phase locking far-field beam convergence effect is optimal [25]. After phase locking, the light field of each unit is coherent, the far field is coherent superposition, and the divergence angle is significantly compressed. The light field of the similar field after phase locking is:

(6) $$E(x,y,0)=E_{0}exp\left(j\phi \right)\sum_{n=0}^{N-1}exp\left(-\frac{(x-nd{)}^{2}}{\omega_{0x}^{2}}-\frac{y^{2}}{\omega_{0y}^{2}}\right)$$

The far-field light field is its Fourier transform, and the main lobe divergence angle is $\theta =\frac{\lambda }{Nd}$. Compared with $\theta =\frac{\lambda }{d}$ without phase locking, the divergence angle is compressed to 1/N, and the beam quality is greatly improved, achieving both high power and high beam quality.

High-quality phase locking needs to meet a series of stringent conditions. Each light-emitting element must work in the fundamental transverse mode state to suppress high-order mode interference, which is a prerequisite for achieving high-visibility interference. At the same time, the thermal stress of the package will cause the optical axis of the light-emitting element to deviate from the straight line, that is, the so-called ‘smile’ effect [26], which seriously destroys the coupling efficiency. Therefore, the design of a low smile array is the key to engineering. In terms of external cavity design, V-shaped or closed V-shaped cavity structures are widely used. They can provide stronger mode discrimination, select in-phase supermodels without spatial filters, and narrow spectral linewidth [27].

The laser beam quality is described by the beam quality factor M^2^, which is a parameter used to quantify the proximity between the actual laser beam and the ideal fundamental mode Gaussian beam. The M^2^ of the ideal Gaussian beam is 1, while the M^2^ of the actual beam is greater than 1. The smaller the value, the better the beam quality. Its theoretical calculation formula is as follows:

(7) $$M^{2}=\frac{\pi \cdot \theta \cdot \omega_{0}}{\lambda }$$

where $\omega_{0}$ is the beam waist radius, λ is the laser wavelength, and θ is the far-field divergence half angle. θ can be obtained by the following formula:

(8) $$\theta \approx \frac{D}{2f}$$

where D denotes the diameter of the beam at the lens and f denotes the focal length of the lens.

The phase-locking efficiency of laser arrays is usually evaluated by the visibility of far-field interference fringes. The calculation formula is as follows:

(9) $$V=\frac{I_{max}-I_{min}}{I_{max}+I_{min}}$$

$\;I_{max}$ is the maximum light intensity, and $I_{mim}$ is the minimum light intensity. The higher the value, the better the contrast of the stripe, which means the higher the phase-locking efficiency.

The output power is another important parameter of the laser. The phase-locking efficiency determines the output power of the main lobe, and the beam quality factor M^2^ determines the concentration of the far-field light intensity. Therefore, by selecting the appropriate external cavity structure and optimizing the laser parameters, the output performance can be significantly improved.

## 3. The Basic Structure of Talbot Cavity in Phase-Locked Laser Diode Array

Early phase-locked research mainly focused on internal coupling. The phase-locked LDA realized by this coupling method has a compact structure and is insensitive to external disturbances. However, in order to ensure the effective coupling between the light-emitting units inside the LDA, the width of the light-emitting units in the array is usually not designed to be large, resulting in a certain limitation of the phase-locked output power and difficulty in meeting higher power requirements. In addition, as the number of light-emitting units increases, the instability of the LDA will also increase. The external coupling method can overcome the shortcomings of insufficient output power of the internally coupled LDA. External cavity mirror coupling is a common external phase-locking method. The external cavity mirror can use ordinary plane mirror or diffraction grating. When working, the LDA is equivalent to running in a composite cavity, and the optical feedback is provided by the external cavity mirror to realize the coupling between the light-emitting units. The structure of this phase-locked system is relatively simple and can obtain good phase-locked effect. However, the working length of the external cavity and the relevant parameters of the LDA need to be carefully selected to ensure the stable operation of the phase-locked system. The Talbot external cavity coupling is based on the Talbot effect in optics, that is, when a uniform plane wave irradiates an infinitely long object with periodic transmittance, the image of the object will be presented at the Talbot distance. Based on this effect, the Talbot cavity is designed: by placing an external cavity mirror at a suitable cavity length, a high loss mechanism of in-phase mode or anti-phase mode is introduced to achieve mode selection.

### 3.1. Ordinary Talbot Cavity

**Figure 2:**
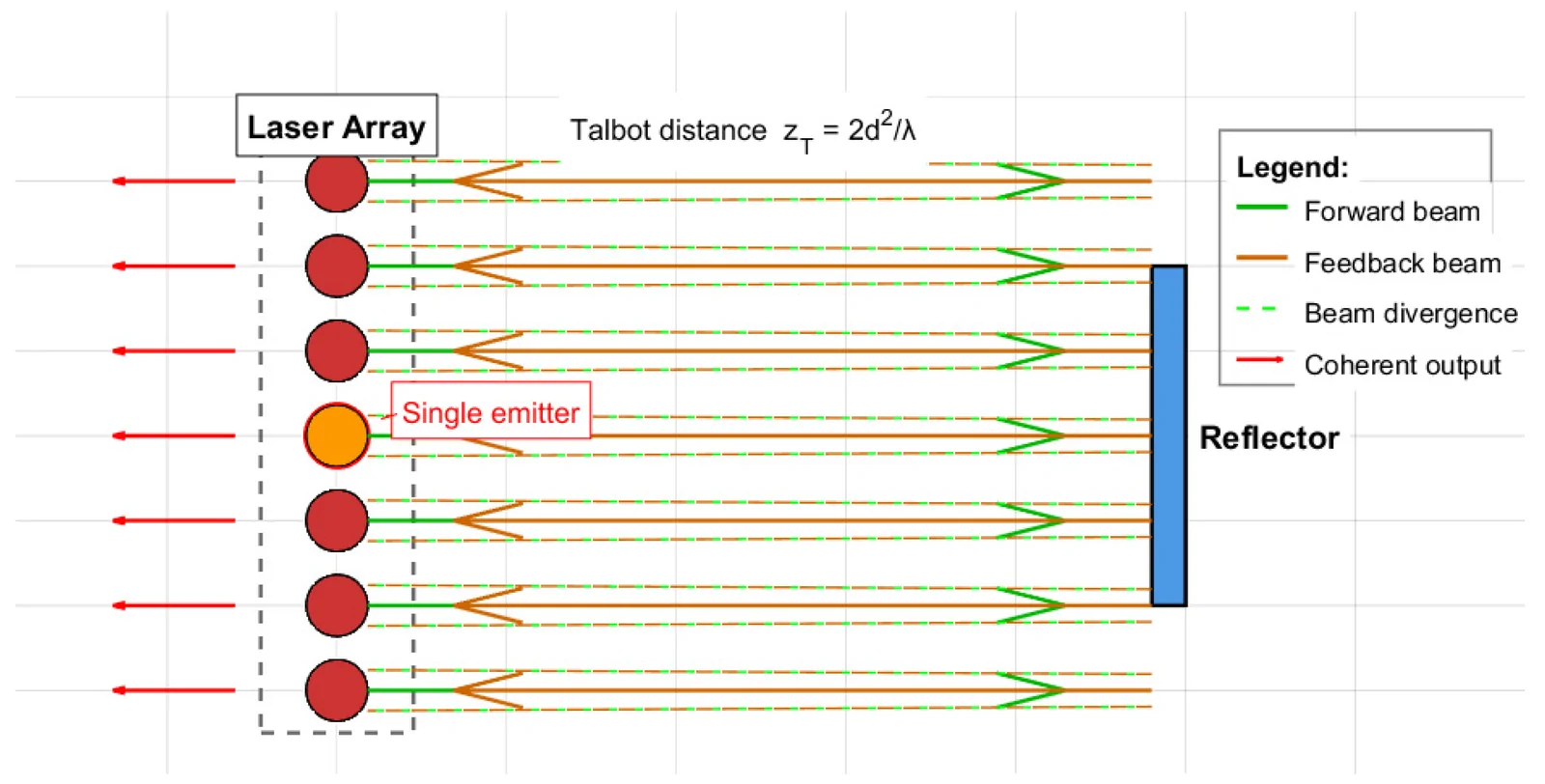
Ordinary Talbot cavity structure in the laser array.

The ordinary Talbot cavity is usually composed of three core parts: periodic laser array, free propagation space and planar feedback mirror. The light-emitting units in the laser array are arranged at equal intervals. The laser beam emitted from each unit of the array with a period of d_0_ propagates forward in free space. After the Talbot distance Z_T_ = 2d^2^/λ, due to the diffraction effect, the periodic light field distribution of the array will spontaneously reproduce as its own self-imaging. A plane mirror is accurately placed at the self-imaging plane, and the mirror feeds the light field path containing the phase information of each unit back to the array [28]. The feedback is not a simple independent unit feedback, but through diffraction coupling, the light fields of all units interact in the cavity, forcing each unit to lock at the same wavelength and maintain a fixed phase relationship, thereby achieving phase locking and coherent beam combining. Finally, the coherently superimposed laser can be output from the transmission end of the mirror or the other end of the array. The core advantage of the ordinary Talbot cavity is to use the self-imaging effect to achieve passive coherent synthesis of multiple light-emitting units. The structure is simple and does not require additional wavelength tuning elements [28]. The ordinary Talbot cavity structure in the laser array is shown in Figure 2.

### 3.2. V-Shaped Littrow–Talbot Cavity

**Figure 3:**
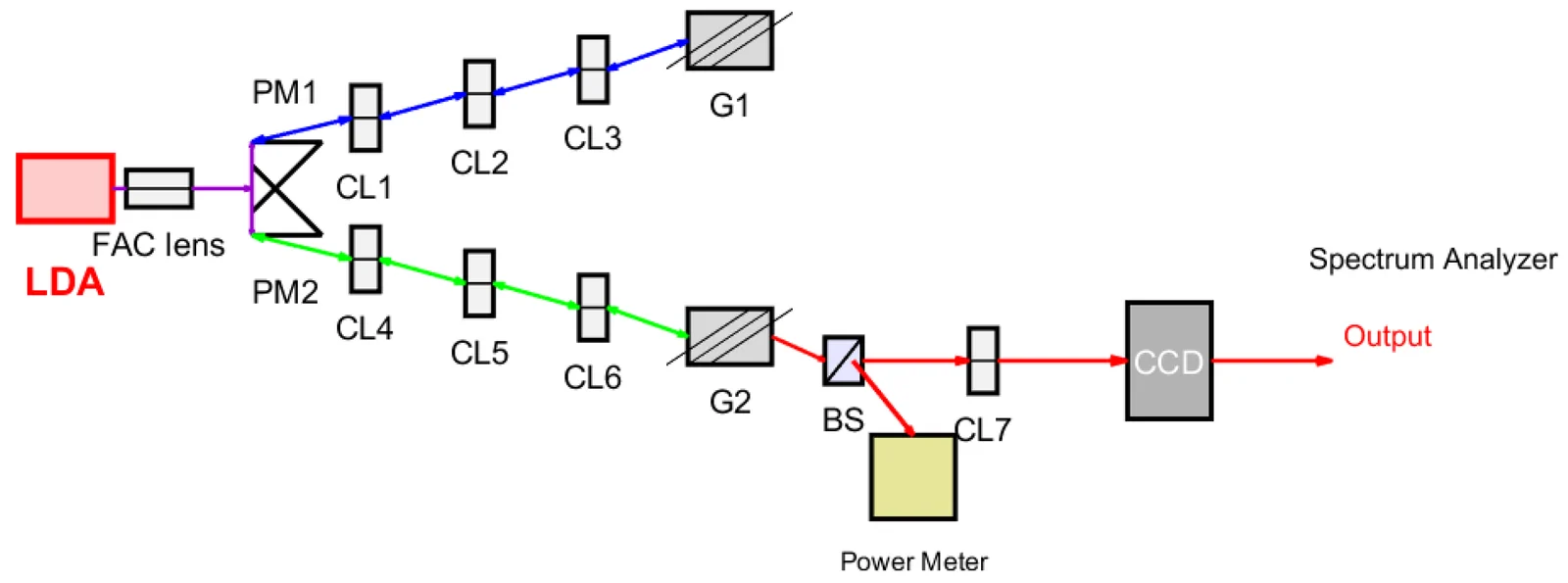
V-shaped Littrow–Talbot cavity structure.

Although the ordinary Talbot cavity can realize the diffraction coupling between laser diodes and lock the phase through the self-imaging effect, it lacks effective suppression of the multi-transverse mode oscillation of a single wide-area laser itself, and the wide-area laser is difficult to obtain high beam quality due to its weak transverse mode selection ability caused by its wide emission size. The V-shaped Littrow–Talbot cavity divides the laser beam into two channels: feedback and output. The diffraction grating is placed in the feedback optical path in the form of Littrow, and the off-axis optical feedback is provided by the first-order diffraction of the grating, so as to selectively enhance a specific transverse mode and suppress other transverse modes, so as to realize the single transverse mode operation of a single-laser diode. At the same time, the V-shaped geometry provides a path for optical coupling between array elements [27]. In addition, the grating configured by Littrow also provides a narrow-band wavelength selection capability, which narrows the spectral linewidth of several nanometers in free operation, and can achieve a tuning range of several nanometers by adjusting the grating tilt angle. The closed V-shaped structure with double grating feedback further improves the quality factor and spectral selectivity of the external cavity, increasing the far-field visibility from 80% to more than 97% and narrowing the spectral width to 0.07 nm [29]. When the grating is located at half Talbot distance, the global coupling and phase locking of all elements of the array can be realized, and the far-field pattern switching between in-phase mode and out-of-phase mode can be realized by adjusting the grating inclination angle. The V-shaped Littrow–Talbot cavity structure is shown in Figure 3 [29].

In a V-shaped Littrow–Talbot cavity, the transmission path of the laser forms a precise closed feedback loop. After the laser is emitted from the front surface of the anti-reflection (AR) coating of the wide-area LDA, its fast axis is first collimated by a telescope system consisting of a fast-axis collimator (FAC) lens and a cylindrical lens to compress the ‘smile’ effect. Subsequently, the beam is divided into left and right channels by a pair of prismatic reflectors to form a V-shaped structure. In the right feedback path, the beam passes through a pair of confocal cylindrical lenses (CL), CL1 and CL3, and the near-field distribution of the laser array is imaged on the focal plane of CL3. The grating (G) G1 is placed at a half-Talbot distance from the focal plane and works under the Littrow configuration. As an end mirror and wavelength selector, G1 accurately feeds the first-order diffracted light that satisfies the Littrow condition back to the laser array along the original path. At the same time, the Talbot self-imaging effect is used to realize the diffraction coupling between the transmitting units in the array, which is the key to achieving phase locking. In the left feedback path, the beam is imaged to the surface of the grating G2 after passing through the FAC lens, CL5, and confocal CL4 and CL6. G2 also works in the Littrow configuration, but it also performs the functions of an output coupler: it feeds back part of the light to the laser array in the form of the first-order diffraction and couples most of the remaining light out of the output cavity to form a useful laser output of the system. Finally, the output beam is converged by the cylindrical lens CL7, and its far-field spot is recorded by the charge-coupled device (CCD) camera. This closed V-shaped cavity composed of two Littrow gratings enhances the feedback, improves the quality factor of the cavity, and enhances the spectral selection and lateral mode control of the laser array, thereby achieving high spatial coherence beam combination.

### 3.3. Monolithic Integrated Talbot Cavity

**Figure 4:**
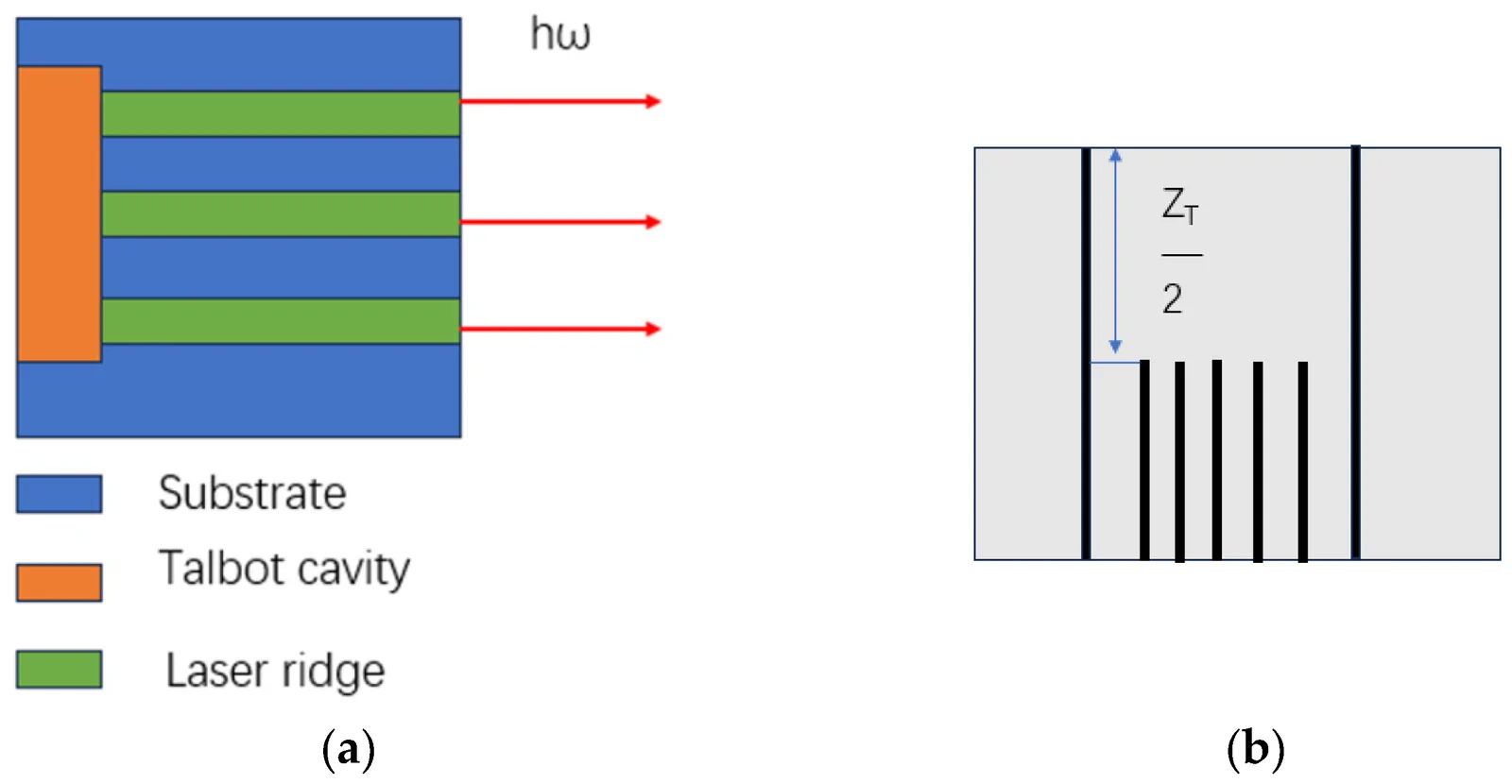
(**a**) Description of the sketch of array device; (**b**) description of Talbot cavity with half Talbot length.

The monolithic integrated Talbot cavity is an on-chip coupling configuration that integrates the Talbot self-imaging phase-locked structure with a semiconductor laser array on the same epitaxial chip. It completely eliminates free space and external mirrors, achieving on-chip diffraction coupling and phase locking through waveguides, cavity facet reflection, or buried gratings. This significantly enhances system compactness, thermal stability, and anti-interference capability, making it a key technological path towards on-chip integrated coherent light sources [30,31]. The core of this structure lies in the semiconductor gain array, where on-chip resonant regions satisfying Talbot or fractional Talbot distances are directly etched or fabricated within the chip [32]; by utilizing waveguide confinement to restrict the propagation of the optical field, the light emitted by the array elements completes periodic self-imaging and feedback coupling within the chip, forcing phase synchronization among the elements and preferentially selecting in-phase supermode output [33]. To further suppress the competition between high-order supermodels and multiple longitudinal modes, a first-order buried DFB grating, absorbing boundary, or mode filtering structure is often employed to ensure stable single-mode operation and high phase-locking visibility over a wide dynamic range. Its core advantages lie in the absence of external optical components, minimal assembly errors, low thermal crosstalk, compatibility with silicon photonic platforms, and the retention of the high robustness of passive phase locking due to the Talbot effect, making it suitable for large-scale arrays and high spectral purity applications. The basic structure of a monolithically integrated Talbot cavity is shown in Figure 4 [34].

The optical field generated by stimulated emission from the laser array unit propagates along the waveguide axis. A portion of the light is directly coupled out through the front cavity facet, while the remaining part propagates backward into the integrated Talbot cavity region at the rear end. The optical field entering the Talbot cavity undergoes multi-slit diffraction and interference due to the periodic distribution of the array, forming a self-image at a distance of half the Talbot distance from the array. After reaching the rear reflective facet of the cavity, the optical field is reflected and propagates back along the original path, passing through the Talbot cavity region again and forming a secondary self-image on the array plane. The phase-locked optical field returns to the laser gain region for amplification, repeating the above-mentioned intra-cavity cycle of “emission-diffraction-reflection-self-imaging” to form stable coherent oscillation. Finally, a high-brightness, near-diffraction-limited coherent beam is output from the front cavity facet.

Comprehensive comparison of three types of Talbot phase-locked cavities: The traditional planar mirror Talbot cavity has a simple structure and is easy to build. It only relies on Talbot diffraction to achieve global coupling, but the lateral mode screening ability is limited, the spectral line width is difficult to compress, and it is less used alone; the V-shaped Littrow–Talbot cavity combined with grating frequency selection and Talbot diffraction coupling can synchronously realize single transverse mode and narrow linewidth output, and the supermode control is flexible, but its free space optical path has large volume and poor assembly fault tolerance. The monolithic integrated Talbot cavity integrates the diffraction coupling structure into the chip to avoid the installation and adjustment error of discrete components. The system stability and integration are optimal, but there are short boards with narrow wavelength adjustment range and high difficulty in process preparation. The three types of cavities all rely on Talbot self-imaging to complete the global passive phase locking of the array, but they have their own trade-offs in coupling loss, mode control, spectral characteristics and engineering adaptability. They can be selected differently according to output power, beam quality and integration requirements.

In today’s research, in addition to the above-mentioned ordinary Talbot cavity and V-shaped Littrow–Talbot cavity, researchers will add other devices or use other composite Talbot cavities according to experimental requirements. For example, Bo Liu et al. [35] used a compact V-shaped external Talbot cavity and volume Bragg grating feedback to obtain the best results of phase-locked large-area laser diodes.

## 4. The Research Progress of Talbot Cavity in Phase-Locked Laser Diode Array

The Talbot cavity is based on the self-imaging effect of the periodic light field. The external cavity mirror is accurately placed at the Talbot distance so that the light-emitting units of the laser array establish a fixed phase relationship through diffraction coupling, thereby passively achieving phase locking and coherent beam combining. It effectively overcomes the limitations of the internal coupling method in power expansion and mode stability and provides a simple and reliable solution for the high brightness and high coherence output of the diode laser array. Therefore, the Talbot external cavity is one of the most commonly used and classical structures in the study of passive phase locking of laser arrays [36]. The following will introduce the research progress of the Talbot cavity in phase-locked LDA.

### 4.1. Experimental Research Progress

In 2008, Liu B et al. [27] first used a novel V-shaped external Talbot cavity to achieve high-quality phase locking of a high-power wide-area LDA. The array consists of 10 emitting units, with a period of 200 μm and a wavelength of 808 nm. The maximum phase-locked output power reaches 9 W, achieving stable in-phase supermode operation. More than 60% of the total power is concentrated in the main far-field lobes near the diffraction limit.

In 2010, Liu B et al. [29] achieved high-quality phase locking of a high-power wide-area LDA using a closed V-shaped external Talbot cavity. The array consists of 10 emitting units, with a period of 200 μm and a wavelength of 808 nm. Thanks to the closed cavity structure, the maximum phase-locked output power reaches 9.3 W, and more than 65% of the total optical power is concentrated in the central near-diffraction-limit lobe.

In 2013, Liu B et al. [37] used an improved V-shaped external cavity Talbot cavity to achieve high-quality phase locking of a high-power wide-area LDA and obtained near-diffraction-limit beam quality and high electro-optic conversion efficiency. The array consists of 10 transmitting units, the array period is 200 μm, the wavelength is 800 nm, the visibility is 99% at 5 A current, the visibility is 95% at 14 A, and the conversion efficiency is 19%.

In 2016, Tradonsky C et al. [38] used a degenerate external cavity combining Talbot diffraction and Fourier filtering to achieve efficient, controllable, and high-fidelity phase locking of a large number of laser arrays. The experiment realized the phase locking of about 450 square arrays, about 1050 triangular arrays, and about 700 honeycomb arrays, with a wavelength of 1064 nm. The phase-locking efficiency of the triangular array at 3/4 Talbot length is about 25% higher than that at 1/2 Talbot length, and the honeycomb array is about 43% higher.

In 2017, Bo M et al. [34] used a monolithic integrated Talbot cavity to explore a new phase-locked array scheme to improve the output power of quantum cascade lasers and obtain high beam quality. The final experiment shows that the laser array composed of six transmitting units has a wavelength of 4800 nm, a coupling efficiency of about 83%, and a maximum peak power of about 4 W. Compared with a single tube, the peak power is increased by more than 5 times.

In 2018, Liu B et al. [35] used a compact V-shaped external cavity Talbot cavity with a volume Bragg grating as a feedback element to achieve coherent combination of a high-power wide-area LDA while maintaining a compact cavity. Finally, the experiment is realized: in the array composed of 10 transmitting units, the array period is 200 μm, the laser wavelength is 800 nm, the far-field visibility is >94%, and the output power is 4.8 W when the current is 11 A.

In 2021, Kopp V I et al. [31] proposed an all-solid-state passive coherent combining scheme, which realized the passive coherent combining of 61 fiber channels in a monolithic integrated Talbot cavity. The laser wavelength is 2000 nm, and the central intensity is 8 times higher than that of incoherent superposition. The measured far field is the secondary side lobe distribution of the central blazed main lobe, and the output power of more than 200 W is realized in the experiment.

In 2022, Xu Y et al. [39] used a monolithic integrated Talbot cavity to obtain power amplification while maintaining high beam quality through a phase-locked array combined with a Talbot cavity. Finally, the experiment is realized: in the array composed of five transmitting units, the period is 220 μm, the frequency is 4.3 THz, the central main lobe is very strong, and the weak lobes on both sides are symmetrical. The average power amplification efficiency is 78%, and the peak power is 359 mW.

In 2022, Nyuaupane P R et al. [40] used a V-shaped Talbot cavity to solve the problem of high-power blue LDA spectral line width and poor coherence. Through the design of a V-shaped external cavity, high power and narrow line width coherent beam combination was realized. Finally, the array is composed of 23 transmitting units, with a period of 400 μm and a wavelength of 447 nm. The line width is narrowed to 20–50 pm, and the output power reaches 11.8 W.

In 2023, Xu Y et al. [41] adopted a monolithic integrated Talbot cavity to realize phase locking of a multi-unit terahertz quantum cascade laser array and completed phase synchronous coherent beam combining based on the Talbot diffraction effect. The laser array composed of six transmitting units has a period of 220 μm, a frequency of 4.3 THz, and a maximum pulse output power of 359 mW at 13 K temperature, achieving stable fundamental mode high-brightness phase-locked output.

In 2024, Nyaupane P et al. [42] used a compact V-shaped semi-Talbot external cavity to experimentally demonstrate the phase locking and coherent combining of high-power, wide-area blue LDA for the first time. Finally, the experiment is realized: in the array composed of 19 transmitting units, the period is 400 μm, the wavelength is 447 nm, and the far-field visibility reaches 87.8%. The far-field pattern shows clear interference fringes, and the peak output power reaches 18.8 W.

In 2024, Xu Y et al. [43] used a monolithic integrated Talbot cavity to realize the phase locking of the array through the Talbot cavity and combined with the first-order buried distributed feedback (DFB) grating to achieve single-mode operation, thereby obtaining a single-mode coherent light source with high brightness and high beam quality. Finally, the experiment is realized: in the array composed of five transmitting units, the period is 220 μm, the frequency is 4.3 THz, and the single-mode power amplification factor is 4.9 times. At 13 K temperature, a single-mode pulse output of 108 mW is achieved.

In 2025, Nyaupane P et al. [44] used a V-shaped semi-Talbot external cavity equipped with a surface grating to study the dynamic path of a high-power, highly non-uniform low-filling-factor blue diode array to achieve phase locking. Finally, there are 24 emitting units to obtain enough feedback light and generate stimulated radiation, and the wavelength is 443 nm. When operating at low power, the far-field visibility is about 56%. When operating at high power, the far-field visibility is about 49%. The peak power of in-phase supermodel phase locking is 10.5 W, and the peak power of anti-phase supermodel phase locking is 9.9 W.

In 2025, Shi J et al. [45] designed a monolithic integrated Talbot cavity for antimonide semiconductor lasers and realized the coherent emission of phase-locked laser arrays. The 15-element and 23-element arrays are fabricated in the experiment, the period is 220 μm, and the wavelength is 2000 nm. The maximum output power is 0.80 W and 1.14 W under continuous wave operation, respectively. The far-field pattern shows a strong central main lobe from the threshold to the full power current, indicating that the device works stably in the fundamental transverse mode.

The Talbot cavity uses self-imaging to achieve passive phase locking without active control. It has a simple structure and strong robustness and is suitable for large-scale integration. Table 1 summarizes the experimental research progress of the Talbot cavity in phase-locked laser arrays in recent years.

Early Talbot cavity phase-locked laser systems primarily relied on the Talbot self-imaging effect, achieving passive phase locking by setting specific propagation distances in an external cavity to enable interference coupling among the emitted beams from individual elements of an LDA. Before 2008, research mainly focused on fundamental and 1/4 Talbot cavities. The fundamental Talbot cavity typically employed integer multiples of the Talbot distance, allowing the periodic image of the array to perfectly overlap with itself, thereby enabling phase locking. In contrast, the 1/4 Talbot cavity exploited fractional Talbot effects, where at one-quarter Talbot distance, images of array elements undergo lateral displacement and interfere constructively, favoring the selection of specific supermodes—particularly in-phase modes—and thus producing output beams approaching the diffraction limit. Leger first systematically reported in 1989 an experimental demonstration of transverse mode control for AlGaAs laser arrays using a Talbot cavity, verifying the feasibility of this approach. Subsequently, Waarts et al. demonstrated in 1991 a GaAlAs array based on a 1/4 Talbot cavity, achieving high-power, nearly diffraction-limited coherent output under continuous-wave operation, marking a representative achievement in early Talbot cavity phase-locking technology. These works established the basic framework of Talbot cavity phase locking; however, cavity length was generally determined by the Talbot distance, resulting in relatively fixed and bulky overall structures.

Since 2008, researchers both domestically and internationally have increasingly explored compact and miniaturized configurations, such as V-shaped external-cavity Talbot cavities, half-Talbot cavities, and monolithically integrated Talbot cavities, continuing to advance research on phase locking and coherent beam combining for LDAs.

The research system has gradually expanded from conventional wide-area LDAs to quantum cascade, terahertz, blue and antimonide mid-infrared lasers. The array configuration includes one-dimensional linear, square, triangular and honeycomb configurations. The unit scale has been increased from more than ten units to thousands of units. The filter feedback elements, such as volume Bragg grating, Littrow surface grating and buried DFB grating, are introduced in the study. It is proved that the 3/4 Talbot cavity length can significantly improve the phase-locking efficiency of the array compared with the 1/2 Talbot cavity length [31]; at the same time, with the help of a V-shaped semi-Talbot external cavity, the nonlinear dynamic characteristics, such as chimera state and cluster state, in the coherent evolution process of a blue light array are revealed [44]. In terms of experimental performance, the far-field visibility of the array is up to 99%, and the beam quality is close to the diffraction limit [37]; the linewidth of the blue light array can be compressed to 20–50 pm [40]. The output power continues to break through, and the peak power of the blue light array is up to 18.8 W [42]. The power of the quantum cascade laser array is more than 5 times higher than that of the single tube [34]. The output power of the fiber channel after passive coherent combining is over 200 W [31]. Terahertz and mid-infrared antimonide arrays also achieve coherent output from hundreds of milliwatts to watts, which can stably maintain the in-phase locking and single-mode operation of the fundamental supermodel in a wide operating range and low-temperature environment [39,41,43,45]. In summary, the Talbot cavity phase-locking technology has become a reliable technical approach to achieve high power, high beam quality and narrow linewidth coherent combining of multi-band laser arrays, which provides an experimental basis and technical support for the engineering application of high-performance laser sources.

### 4.2. Research Progress of Numerical Simulation

In 2020, Gavrielides A et al. [46] established a nonlinear dynamic theoretical model for a monolithic Talbot cavity globally coupled quantum cascade laser array. Through the equivalent ring laser modeling and numerical simulation, the model can accurately calculate the stability of each supermodel, capture the unsteady behavior of bifurcation to pulse, and coincide with the existing research under the small signal gain limit, which provides a reliable theoretical tool for the design of a high-brightness mid-infrared coherent light source.

In 2022, Wang X F et al. [47] found that in-phase mode phase locking can be realized by injecting half-cycle face-to-face into the Talbot cavity, with a filling factor of 0.08~0.2, single longitudinal mode frequency locking can be realized by 0.09~0.25, and the robustness of phase locking is the strongest when the filling factor is about 0.1.

In 2023, Li W et al. [48] designed a fractional-order Talbot external cavity for annular VCSEL arrays and achieved in-phase supermodel selection by adjusting the cavity length. Based on the 24-element toroidal array, the far-field distribution of in-phase mode is obtained. The Strehl ratio of the in-phase mode is three orders of magnitude higher than that of the anti-phase mode, which provides a potential solution for the preparation of Bessel–Gaussian beams.

In 2024, Li W et al. [49] introduced a fractional-order Talbot cavity into a ring VCSEL array to achieve in-phase supermodel operation by suppressing high-order supermodels. Based on the example of a 24-element toroidal array, the regulation of the cavity length on the coupling coefficient and threshold gain is demonstrated, and the far-field distribution of the in-phase mode is obtained, which provides a potential scheme for the preparation of Bessel–Gaussian beams.

In 2025, Jun Qi et al. [50] designed a hybrid resonator diode laser array for high-power coherent semiconductor lasers and realized self-organized phase locking by using the on-chip Talbot effect. By optimizing the structural parameters through numerical simulation, it is measured that the peak energy ratio of the far-field center increases from 24.1% to 42.3%, the lateral divergence angle is significantly compressed from 4.44° to 0.34°, and the scalability of the scheme on the 19-element array is verified.

In summary, significant progress has been made in the numerical simulation of semiconductor laser arrays based on the Talbot effect from 2020 to 2025. Researchers have continuously enriched the theoretical model and structural design from monolithic Talbot cavity, mutual injection structure, fractional-order external cavity to on-chip hybrid resonant cavity. The simulation work successfully reveals the key mechanisms such as supermodel stability, phase-locked robustness, and in-phase mode selection and achieves breakthroughs in performance indicators such as far-field energy concentration and divergence angle compression. In 2026, the related research [51] further expanded the dimension of array simulation, realized the electrical–optical joint simulation through the large-signal equivalent circuit model of a high-power diode array, quantitatively characterized the non-uniformity of unit current caused by array parasitic parameters, explained the inherent law of phase-locked instability induced by electrical disturbance, and provided important theoretical support and optimization direction for mid-infrared and visible light coherent light sources with high brightness and high beam quality.

## 5. Conclusions and Prospects

The Talbot cavity has become a classical technical path for LDAs to achieve phase locking and coherent beam combining by virtue of its passive phase-locking mechanism based on the periodic light field self-imaging effect. Reviewing the research progress in recent years, there are several significant development trends in this field. First, from the early phase-locking verification of a few units, it is gradually extended to hundreds or even thousands of units of large-scale array phase locking, which proves the scalability of the Talbot cavity in power expansion. Secondly, the external cavity structure evolves from the basic planar mirror Talbot cavity to V-shaped Littrow–Talbot cavity, degenerate external cavity, monolithic integrated Talbot cavity and other forms. The mode selection, spectral line width, integration and output power are optimized in different dimensions. Third, the application band extends from the traditional near-infrared to blue, mid-infrared and terahertz bands, showing the universality of Talbot phase-locked technology in various gain media.

Despite significant progress, this field still faces several common challenges: how to maintain high visibility phase locking in larger arrays, how to effectively suppress thermal crosstalk and the ‘smile’ effect under high-power operation, and how to achieve on-chip integration compatible with silicon optical platforms. In the future, Talbot cavity phase-locked technology is expected to expand to a wider spectral range. With the help of an intelligent control algorithm, real-time monitoring and dynamic optimization of the phase-locked state can be realized, and new coherent beam synthesis schemes can be developed by using new physical effects such as chimera state, so as to provide continuous impetus for the practical application of high-brightness semiconductor lasers.

## Figures and Tables

**Table 1:** The latest research progress of Talbot cavity phase-locked laser arrays (“a” represents a basic number indicating the locking efficiency).

Type	Wavelength or Frequency	Number of Lasers	Period	Far-Field Visibility	Output Power	Year
V-shaped	770 nm	49	200 μm	——	9 W	2008 [27]
V-shaped	808 nm	47	200 μm	——	9.3 W	2010 [29]
V-shaped	800 nm	10	200 μm	99% (current is 5 A)	——	2013 [37]
				95% (current is 14 A)		
Degenerate cavity	1064 nm	450 (square array)	——	a (Lock-in efficiency)	——	2016 [38]
		1050 (triangle array)	——	1.25 a	——	
		700 (honeycomb array)	——	1.43 a	——	
Monolithic integrated	4800 nm	6	——	83% (coupling efficiency)	4 W	2017 [34]
V-shaped	800 nm	10	200 μm	>94% (current is 11 A)	4.8 W	2018 [35]
Monolithic integrated	2000 nm	61	——	——	200 W	2021 [31]
Monolithic integrated	4.3 THz	5	220 μm	——	359 mW	2022 [39]
V-shaped	447 nm	23	400 μm	——	11.8 W	2022 [40]
Monolithic integrated	4.3 THz	6	220 μm	——	359 mW	2023 [41]
V-shaped	447 nm	19	400 μm	87.8%	18.8 W	2024 [42]
Monolithic integrated	4.3 THz	5	220 μm	——	108 mW	2024 [43]
V-shaped	443 nm	24	——	56% (under low power)	10.5 W	2025 [44]
				49% (under high power)	9.9 W	
Monolithic integrated	2000 nm	15	220 μm	——	0.8 W	2025 [45]
		23		——	0.8 W	

## Data Availability

No new data were created or analyzed in this study. Data sharing is not applicable to this article.
